# Cell and Gene Therapy Approaches for Cardiac Vascularization

**DOI:** 10.3390/cells1040961

**Published:** 2012-11-05

**Authors:** Ludovic Melly, Stefano Boccardo, Friedrich Eckstein, Andrea Banfi, Anna Marsano

**Affiliations:** 1 Cell and Gene Therapy, Department of Biomedicine and Department of Surgery, Basel University Hospital, Basel 4031, Switzerland; Email: lmelly@uhbs.ch; 2 Cardiac Surgery, Department of Surgery, Basel University Hospital, Basel 4031, Switzerland; Email: feckstein@uhbs.ch; 3 Department of Robotics, Brain & Cognitive Sciences, Istituto Italiano di Tecnologia, Genova, 16163 Italy; Email: Stefano.Boccardo@iit.it

**Keywords:** angiogenesis, VEGF controlled release, cell therapy, gene therapy, cell-based gene therapy, cardiac tissue engineering, cardiac ischemia

## Abstract

Despite encouraging preclinical results for therapeutic angiogenesis in ischemia, a suitable approach providing sustained, safe and efficacious vascular growth in the heart is still lacking. Vascular Endothelial Growth Factor (VEGF) is the master regulator of angiogenesis, but it also can easily induce aberrant and dysfunctional vascular growth if its expression is not tightly controlled. Control of the released level in the microenvironment around each cell *in vivo* and its distribution in tissue are critical to induce stable and functional vessels for therapeutic angiogenesis. The present review discusses the limitations and perspectives of VEGF gene therapy and of different cell-based approaches for the implementation of therapeutic angiogenesis in the treatment of cardiac ischemia.

## 1. Introduction

Ischemic heart disease is one of the most frequent causes of death worldwide [1]. The impact of cardiovascular diseases on the health care system is estimated to grow as the population ages. Coronary revascularization strategies (such as percutaneous coronary intervention and coronary artery bypass grafting) currently provide the best treatment for patients with coronary artery disease. Revascularization procedures mainly aim to improve cardiac function and reduce the risk of myocardial infarction (MI). Some patients are not suitable candidates for surgery or angioplasty to restore the vascular flow distal to a stenosis because of age, comorbidities or unfavorable vascular anatomy, and are not adequately treated despite optimal pharmacologic therapy. A pro-angiogenic therapy that stimulates micro-vascular growth might also be beneficial in these patients. In fact, while revascularization procedures directly bypass or remove the obstruction in the large conductance arteries, expansion of the micro-vascular bed is capable of inducing the enlargement of upstream collateral arteries through increased shear stress and gap junction-mediated retrograde signaling along vessel walls, thereby restoring perfusion downstream of the occlusion by way of a biological bypass [2,3]. Several gene and cell therapy approaches showed potential benefit and cardiac function improvement in preclinical studies [4]. However, a suitable adjuvant treatment for chronic cardiac ischemia, capable of providing a long-term and, at the same time, safe and efficacious angiogenic stimulus, still needs to be identified. This review aims at briefly overviewing the basic mechanisms of angiogenesis and approaches to translate these in therapeutic strategies for the treatment of cardiac ischemia, highlighting their advantages and limitations. In particular, it will focus on the biology of Vascular Endothelial Growth Factor (VEGF), specifically of the most commonly used isoform, *i.e.*, VEGF_165_, and its implications for pro-angiogenic strategies. These approaches find application in two related fields within the treatment of cardiac ischemia: (i) direct myocardial revascularization, and (ii) vascularization of engineered cardiac patches. The first approach aims at restoring the blood supply to the chronically ischemic border zone of infarcted tissue, consisting of still viable but dysfunctional (hibernating) myocardium. The second approach aims at improving the function of damaged myocardium by implantation of a contractile cardiac patch: in this setting, rapid vascularization *in vivo* is crucial to ensure the survival and function of the seeded progenitors inside the thick tissue-engineered construct.

## 2. The Microenvironmental Regulation of Angiogenesis

VEGF is the master regulator of vascular growth and it is the most specific single factor capable of starting the complex cascade of events leading to angiogenesis. Inactivation of VEGF during development results in embryonic lethality and myocardial defects [5,6], whereas VEGF delivery has been shown to induce new vascular growth and improved cardiac function in preclinical models of myocardial infarction [7]. Angiogenesis is the growth of new microvessels starting from pre-existing ones. Extravasation of plasma occurs immediately after VEGF stimulation, as a consequence of increased vessel permeability by loosening of the endothelial junctions and by detachment from the vascular wall of mural cells, *i.e.*, pericytes and smooth muscle cells. The extravasated plasma provides a provisional matrix that supports endothelial cell migration. VEGF stimulates quiescent endothelial cells to become activated, causing them to migrate and proliferate. The orderly regulation of this morphogenic process is controlled by Notch signaling [8,9]. In fact, VEGF binds to the extracellular matrix and forms gradients in the microenvironment around each producing cell [10]. The first cells that are activated by VEGF become specialized tip cells, which do not proliferate and are not involved in the formation of lumen structures, but rather sense the gradient of VEGF in the microenvironment and migrate towards it, thereby initiating the sprouting process. In response to VEGF, tip cells upregulate expression of Delta-like-4 (Dll4), which activates Notch-1 signaling on the neighboring endothelial cells and instructs them to acquire a stalk cell phenotype, instead. Contrary to tip cells, stalk cells respond to the total concentration of VEGF by proliferating rather than migrating and form the “stalk” of the new vessel behind the sprouting tip. Vascular maturation, which involves the return of endothelial cells to quiescence and independence from continued VEGF signaling, requires a complex crosstalk between the endothelium and pericytes (reviewed in [11]), which are recruited by Platelet-Derived Growth Factor-BB (PDGF-BB), produced by activated tip cells [8]. An active role in vascular maturation is also played by recently identified populations of myeloid cells [12], which are recruited by signaling through the VEGF and Semaphorin receptor Neuropilin-1 and produce a host of pro-maturation paracrine factors, including PDGF-BB, Angiopoietin-1 and TGF-β1 [13].

Over the last decade, studies with different delivery platforms have identified key requirements to be considered in order to exploit the therapeutic potential of VEGF gene expression, regarding dose, distribution and duration. In fact, uncontrolled over-expression of VEGF has been shown to induce the growth of angioma-like vascular tumors in a variety of tissues, such as skeletal muscle [14,15,16], subcutaneous fat [15], liver [17] and heart [18,19,20]. However, it has been difficult to establish a therapeutic window for VEGF gene delivery, with lower doses of gene therapy vectors being inefficacious and higher ones rapidly causing aberrant vascular growth [20,21], and controlled clinical trials have failed to demonstrate therapeutic efficacy at safe vector doses [22,23]. Taking advantage of a well-characterized cell-based platform for controlled gene delivery, in which clonal populations of transduced myoblasts are used to homogeneously express specific VEGF doses *in vivo*, we found that the therapeutic window of VEGF does not depend on the total dose administered, but rather on its level of expression in the microenvironment around each producing cell [14,24]. In fact, since VEGF remains tightly localized in tissue after being secreted, different growth factor concentrations do not average with each other, even between neighboring muscle fibers, and a few "hotspots" of high expression are sufficient to cause angioma growth even if the total VEGF dose is rather low. However, when a homogeneous distribution of expression levels was achieved by implanting clonal populations in which every cell produced the same amount, it became clear that a wide range of VEGF levels exists that induce only normal, stable and functional capillary networks, and that angiomas are induced only by doses above a discrete threshold level [14]. Furthermore, within the safe range there exists a therapeutic window, whereby too low doses efficiently induce normal angiogenesis, but provide no therapeutic benefit, whereas higher ones induce the growth of normal vessels of larger caliber, which are also effective in forming collateral arteries and restoring blood flow in ischemic tissue [24].

A further key parameter to consider in therapeutic VEGF gene delivery is the duration of expression. In fact, although the morphogenic events leading to the formation of new vascular structures are complete within the first 7 days after VEGF gene delivery [25], the newly induced vessels are unstable and depend on continued VEGF stimulation until about 4 weeks and, if expression is lost before this time, they regress and disappear [14,17,26]. Interestingly, the stabilization of VEGF-induced vessels requires more than just pericyte recruitment, since new capillaries are already fully invested by normal pericytes 7 days after induction [25], but remain VEGF-dependent for a longer time. A better understanding of the molecular and cellular mechanisms regulating this process will be critical in order to design rational strategies for therapeutic angiogenesis.

## 3. Revascularization Strategies for Ischemic Myocardium

Therapeutic angiogenesis strategies aim at restoring effective perfusion of ischemic myocardium in order to improve regional contractility and global cardiac function. Here we will briefly review three broad categories of pro-angiogenic strategies for myocardial ischemia: (i) cell therapy, (ii) gene therapy, and (iii) cell-based gene therapy. Recently identified populations of cardiac progenitor cells hold promise to regenerate lost cardiomyocytes [27]. However, other classes of progenitors, which do not have significant potential for cardiac differentiation, such as endothelial progenitor cells and adult mesenchymal stem cells, have been found to effectively improve the function of infarcted myocardium mainly through pro-angiogenic and anti-apoptotic paracrine effects [28]. Here we will focus on cell therapy approaches for cardiac revascularization rather than regeneration. Gene therapy approaches consists instead in the direct delivery of therapeutic genes in a viral or DNA-based vector, while cell-based gene therapy is a combination of these two approaches based on the delivery of progenitors previously transduced *in vitro* to express the therapeutic gene of interest.

### 3.1. Cell Therapy

For cardiac revascularization, endothelial progenitor cells (EPC) derived from either peripheral or umbilical cord blood or from bone marrow have been often used in clinical trials. In particular, CD34^+^ [29,30] or CD133^+^ [31,32] purified cells have been shown to improve angiogenesis and cardiac function both in animal models and in clinical trials [33]. However, the mechanism or mechanisms by which functional improvement is achieved are controversial. In fact, most evidence points out that so-called EPC are actually not incorporated into new vessels as either endothelial or mural cells, but rather may provide paracrine stimulation of both angiogenesis and tissue protection through the production of as yet not clearly defined combinations of factors [34]. Mesenchymal stem cells (MSC) of different origin, such as from the bone marrow or adipose tissue, have been investigated in several clinical trials as a treatment for the sequelae of myocardial infarction [7]. MSC can release a broad range of factors with pro-angiogenic, anti-apoptotic, anti-inflammatory, immunomodulatory and anti-scarring functions [35]. Through their paracrine effects, MSC have been demonstrated to increase blood vessel growth in numerous *in vivo* models [36,37]. A specific advantage of lipoaspirate-derived expanded MSC and the native stromal vascular fraction cells of adipose tissue is their availability, as they can be easily procured in large quantities with very limited donor site morbidity. Cell therapy offers a very attractive approach for the treatment of cardiac ischemia from a safety and regulatory point of view, thanks to the use of autologous cells and the absence of genetic modification. However, a difficulty in using cell-based therapies lies in the identification and characterization of the specific sub-populations responsible for the therapeutic effect. Furthermore, employed cells produce a variety of growth factors, which are also likely to act synergistically, and this makes it complicated to identify the specific mechanisms that could be targeted to increase therapeutic efficacy. Lastly, a major unsolved issue in this approach is the poor cell retention and survival upon direct intra-myocardial delivery.

### 3.2. Gene Therapy

Plasmid DNA, adenovirus (AV) or adeno-associated virus (AAV) are the most commonly used vectors to deliver VEGF. Plasmid DNA is very easy to produce. However, the gene transfer efficiency *in vivo* is very low and, as the plasmid DNA is gradually destroyed after uptake, the duration of expression is transient, lasting up to a couple of weeks. For these reasons, plasmid vectors have shown a good safety profile, but a low efficiency makes their clinical relevance not clear [38]. Furthermore, plasmid DNA does not intrinsically allow specific delivery to the target tissue of interest, although methods to overcome this limitation by ultrasound-mediated delivery with microbubbles are currently being investigated [39].

The efficiency of gene transfer improves with viral vectors. Adenoviruses can be easily produced at high titers, can accommodate large expression cassettes and can transduce multiple cell types, both proliferating and quiescent. Therefore, AV have been widely used in gene therapy applications [40]. On the other hand, AV produce a very high initial level of gene expression, with a peak few days post-injection, but expression drops rapidly and only lasts 10–14 days because of the strong immune response, which also precludes repeated administration of AV of the same serotype [38].

AAV are small vectors with a limited transgene capacity, but possess many advantageous features. AAV exist in different serotypes with different tropisms for target tissues. In particular serotypes 1, 6, 8 and 9 are very effective to transduce adult skeletal muscle and myocardium [41]. Wild-type AAV mostly remain episomal (about 90% of the vector genomes), but about 10% do integrate into a specific site of the host chromosome 19 (19.13.3-qtr, also called AAVS1). However, since the viral *Rep* protein, which is required for integration, is deleted in AAV-derived vectors, their integration rate is reduced to <1% of the proviral genomes and it is no longer specific for the AAVS1 site [42]. Contrary to AV, AAV are very poorly immunogenic, and therefore they are suitable for long-term gene expression up to several months. Importantly, the kinetics of gene expression is very different from AV, *i.e.*, a lower maximal expression level that is achieved gradually with a delayed onset over a couple of weeks after injection, due to the need to convert the single-stranded DNA viral genome to a double-stranded form before transcription can begin [43]. An issue that can diminish efficacy of AAV-based gene therapy is the presence of pre-exisiting neutralizing antibodies to the capsid proteins [43]. This may be tackled by selecting serotypes for which natural immunity in humans is absent or minimal.

Although several preclinical studies showed positive results, a suitable gene therapy approach providing a long-term, safe and efficacious angiogenic stimulus is still lacking [4,23]. Some of the key factors that determine the effectiveness of gene therapy for therapeutic angiogenesis have been identified as: (i) the efficacy of gene transfer and effective dose achieved *in vivo*, (ii) the duration of the angiogenic stimulus, and (iii) the biological potency of the delivered molecule [44]. The specific requirements regarding the dose and duration of VEGF expression as a single factor, which are challenging to satisfy with direct gene therapy approaches, have been analyzed above. In this respect, recent findings suggest that co-delivery of maturation factors, such as PDGF-BB, which controls pericyte recruitment, or Angiopoietin-1, or factors that are both angiogenic and maturative, like FGF-2, may help to overcome these limitations [44]. In particular, Korpisalo *et al.* showed that the co-injection into rabbit skeletal muscle of two different adenoviruses carrying VEGF-A and PDGF-BB resulted in a prolonged angiogenic response, despite short-term gene expression. However, this effect was mostly mediated via paracrine effect from recruited mononuclear cells rather than increased pericyte coverage [45]. Further, co-delivery of AAV vectors encoding these two factors in a rabbit model of hindlimb ischemia and a pig model of hybernating myocardium significantly increased the therapeutic efficacy of low doses of AAV-VEGF, which were ineffective alone [46]. Recently we found, by complementary gain- and loss-of-function experiments, that the threshold between normal and aberrant angiogenesis is not an intrinsic property of VEGF dose alone, but depends on the balance between VEGF and PDGF-BB signaling. As a consequence, coordinated co-expression of VEGF and PDGF-BB at fixed relative levels through a single bicistronic vector switched the induction of angioma growth to homogeneous normal capillary networks, despite heterogeneous and high VEGF expression levels, and greatly improved functional efficacy of VEGF expression in a model of hindlimb ischemia [47]. Furthermore, we also found that PDGF-BB co-expression accelerated stabilization of vessels induced by heterogeneous VEGF levels, so that 50% of new vessels were VEGF-independent already after 2 weeks, whereas none were stable when VEGF was expressed alone (Reginato and Banfi, unpublished data). In conclusion, these data suggest that coordinated co-expression of VEGF and PDGF-BB is a promising approach to both normalize and stabilize VEGF-induced angiogenesis despite uncontrolled and short-term expression.

Contrary to the widespread effects of VEGF-A, VEGF-B has been recently shown to induce angiogenesis and arteriogenesis specifically in the myocardium, but not in other tissues [48], together with a function in the maintenance of physiological homeostasis in the heart and an anti-apoptotic effect on cardiomyocytes, leading to restoration of perfusion and functional improvement in large-animal models of myocardial infarction [49]. Furthermore, VEGF-B has been shown to have non-angiogenic functions in the direct regulation of cardiomyocyte metabolism and hypertrophy [50,51]. These findings show potential to develop heart-specific angiogenic and cardio-protective therapies.

### 3.3. Cell-based Gene Therapy

**Figure 1:**
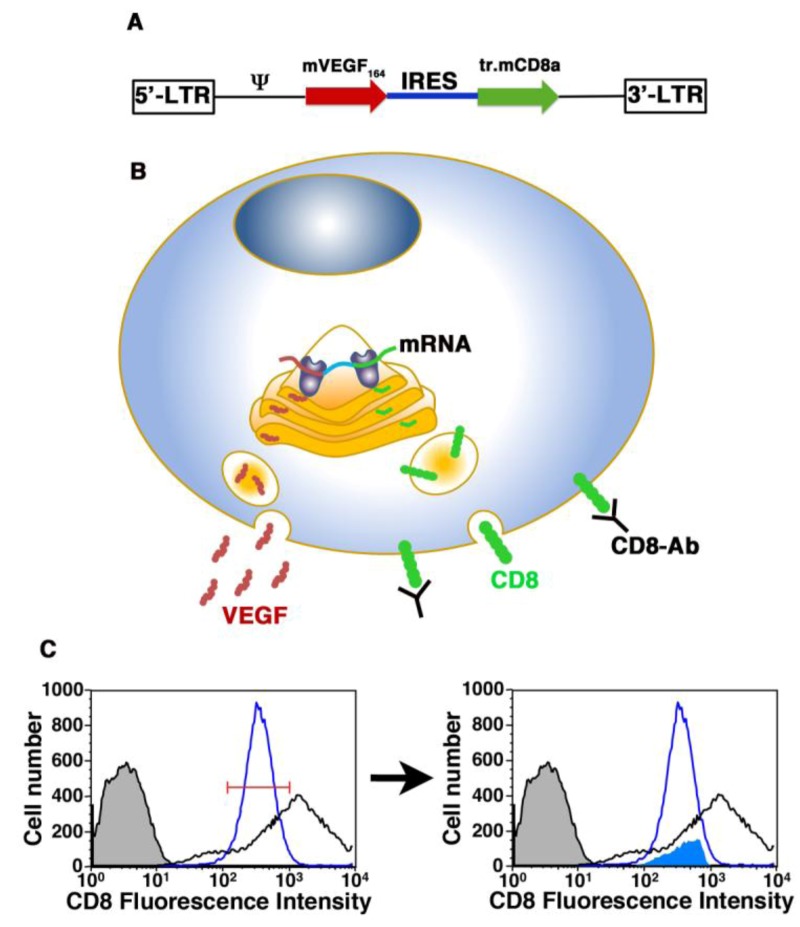
Schematic representation of the FACS-based cell purification technique. (**A**) Structure of the retroviral construct (VICD8) used to co-express VEGF (mVEGF_164_) and a truncated version of CD8 (tr.mCD8a), linked through an Internal Ribosomal Entry Sequence (IRES). (**B**) In transduced cells, each transcribed mRNA molecule contains both sequences, which are then co-translated by classic cap-dependent and IRES-dependent ribosomal attachment, respectively. Therefore, regardless of the amount of expression from each integrated vector copy, the amount of VEGF protein secreted is always proportional to the amount of truncated CD8 on the cell surface, which can be detected by antibody staining (CD8-Ab) and quantified by FACS. (**C**) FACS-based purification of polyclonal populations stably expressing specific levels of VEGF and CD8a. The histogram plot in the left panel represents fluorescence intensity of CD8 staining in transduced myoblast populations measured by flow cytometry. Based on the fluorescence intensity of a reference clonal population (empty blue curve) a specific sorting gate was designed (red segment). This gate was applied to the heterogeneous VICD8 population (empty black curve) to sort one specific subpopulation (shaded blue curve in the right panel) that expressed the same level of VEGF as the reference. Negative control cells are represented by the tinted black curve in both panels. Adapted from Misteli *et al.* [52] and Wolff *et al.* [53]. Graphics by Dr. N. Di Maggio.

The use of cells as factor delivery system has the potential to overcome some of the issues that limit the usefulness of VEGF gene therapy. This approach combines the sustained release of the therapeutic factors, characteristic of gene therapy, with the possibility to exploit the production of synergistic paracrine factors by the delivered progenitors, characteristic of cell therapy. Furthermore, it allows a unique opportunity to control the distribution of microenvironmental expression levels *in vivo*. For this purpose we have recently developed a high-throughput, Fluorescence Activated Cell Sorting (FACS)-based technology to identify and rapidly purify cells expressing a specific desired VEGF level from a heterogeneous population of transduced progenitors. This was achieved by linking VEGF expression to that of a FACS-quantifiable syngenic cell surface marker (truncated CD8a), so that the amount of CD8 on the cell surface would reflect the level of secreted VEGF [52] (Figure 1).

Populations of VEGF-expressing primary myoblasts purified in this way yielded robust, normal and stable angiogenesis both in normal [52] and chronically ischemic [53] skeletal muscle, while angioma growth was completely avoided. Taking advantage of this technology, we could also generate purified populations of bone marrow- and adipose-derived mesenchymal progenitors expressing specific VEGF levels [54]. Recent data we obtained by implanting purified and non-purified VEGF-expressing human adipose tissue-derived MSC in both normal and ischemic myocardium show that only purified progenitors releasing a controlled and safe level of VEGF induced normal and stable angiogenesis, prevented angioma growth and prevented deterioration of cardiac function after coronary artery ligation [55]. However, a still unresolved issue, limiting the efficacy of both cell therapy and cell-based gene therapy approaches, is the poor engraftment of injected cells in the myocardium.

## 4. Vascularization of Engineered Cardiac Tissue

In the past years several cardiac repair and angiogenic strategies have been based on the tissue engineering paradigm [56,57,58,59,60], which implies the seeding of 3D biomaterials with an appropriate cell source: the cells, perfused through the scaffold pores, colonize the biomaterial in its full thickness, and are cultured *in vitro* for a variable amount of time before *in vivo* grafting. The cardiac tissue engineering approach offers several advantages compared to simple cell injections, which lead to very low cell survival and inconsistent graft size [61,62,63]. Indeed, poor cell engraftment is one of the factors underlying the limited benefit observed in the current MSC-based clinical trials [64]. Furthermore, injection-based cell therapies can cause (i) damage to the myocardium, (ii) an undesired inflammatory response, and (iii) localization of injected cells in clusters, which might create an electrical barrier to direct wave propagation [64].

An approach with the potential to overcome these limitations would be to develop a cell-loaded patch that generates contractile tissue with myocardium-like mechanical properties and with the possibility of electromechanical coupling to the host cardiomyocytes. A growing number of cardio-progenitor populations are the subject of current active research to identify the optimal source to generate functional myocardium-like tissue [27]. However, it’s also clear that *in vivo* vascularization of a cardiac patch of clinically relevant thickness will be essential to maintain cell viability and function upon implantation, and to eventually enable electrical coupling and mechanical integration of the patch tissue with the surrounding myocardium. For instance, both alginate- [65] and gelatin- [66] based scaffolds seeded with neonatal rat cardiomyocytes showed some endothelial structures only several weeks after implantation in an ischemic myocardium, and just a limited benefit on the cardiac functionality was achieved.

Other studies used a different approach, pre-vascularizing the engineered cardiac patches by either implantation into the omentum [67], which requires a supplementary surgical procedure, or by co-culturing cardiomyocytes [68,69] or cardiac progenitors [70] with endothelial cells. However, both approaches led to only limited survival and differentiation of the seeded cells. Other strategies that have been explored include the use of proangiogenic scaffolds with a pore architecture designed to facilitate infiltration by blood vessels or the use of VEGF-functionalized scaffolds [71,72]. Overall, despite some promising results, prompt vascularization still remains a fundamental bottleneck towards the generation of tissue-engineered cardiac constructs of clinically relevant size.

Results we recently obtained suggest that the combination of cardiac tissue engineering with cell-based pro-angiogenic gene therapy might provide a novel approach to ensure rapid and effective vascularization of a contractile cardiac patch upon grafting on ischemic tissue. As a proof-of-principle, we generated a millimeter-thick cardiac patch composed of naïve neonatal rat cardiomyocytes (RCM) and murine skeletal myoblasts (SM), genetically modified and FACS-purified to express a specific, safe and effective VEGF level, according to the technology described in Figure 1 [73]. After implantation in a nude mouse model of cardiac infarction, only the co-seeding of VEGF-expressing cells allowed the induction of efficient, normal angiogenesis inside the patch, leading to the survival of both RCM and SM and robust differentiation of RCM into mature myofibers, with a significant improvement in cardiac contractility. The newly induced vascularization was comprised only of physiological networks of capillaries, homogeneous in size and fully mature, *i.e.*, invested by normal pericytes, defined by the characteristic elongated processes and expression of the proteoglycan NG2, but not of smooth muscle actin. This is in agreement with our previous results in other systems showing that controlled VEGF expression is sufficient to induce normal, mature and functional vascular growth without the need for co-expression of maturation factors [14,24,52,53]. Interestingly, vascularization was significantly increased also in the underlying myocardium, where no VEGF-expressing cells were present. It can therefore be envisioned that controlled VEGF expression in a cardiac patch may represent a functional tool with a dual effect, *i.e.* driving effective vascularization both within the engineered patch, leading to progenitor survival, differentiation and function, and in the underlying ischemic myocardium, leading to improved contractility of the under-perfused endogenous tissue.

## 5. Conclusions

Despite significant progress made in the past years, many limitations remain to identify an efficient and safe angiogenic therapy for the treatment of cardiac ischemia. Specific obstacles, which limit the success of individual approaches, might be overcome by combination strategies, as described above. Further, technological advancements in the field of biomaterial science might offer valid alternative strategies that may avoid *ex vivo* genetic cell manipulation. For example, the combination of non-modified progenitors with gene-activated matrices, or smart materials that allow a finely tuned control of both the dose and kinetics of release of recombinant factors, are very appealing approaches for clinical applications. Regardless of the specific technology, a greater understanding of the mechanisms of both induction and maturation of blood vessels will be critical to generate the basic concepts to be translated into the clinical development of rational pro-angiogenic therapies.
